# Comparison among cryoablation, radiofrequency ablation, and partial nephrectomy for renal cell carcinomas sized smaller than 2 cm or sized 2–4 cm: A population-based study

**DOI:** 10.1097/MD.0000000000015610

**Published:** 2019-05-24

**Authors:** Shuai Yan, Wei Yang, Cheng-mei Zhu, Pei-meng Yan, Zhi-chao Wang

**Affiliations:** a Department of Urology; b Department of Diagnostic Imaging; c Department of Clinical Laboratory, Harbin 242 Hospital; d Department of Urology, Harbin Medical University, Harbin, China.

**Keywords:** cryoablation, partial nephrectomy, radiofrequency ablation, renal cell carcinoma

## Abstract

This study aimed to compare radiofrequency ablation (RFA), cryoablation (CRA), and partial nephrectomy (PN) for renal cell carcinoma (RCC) sized ≤2 cm or 2 to 4 cm.

The Surveillance, Epidemiology, and End Results (SEER) database was used to identify 17,234 patients diagnosed with T1aN0M0 RCC from 2004 to 2015. Overall survival (OS) and cancer-specific survival (CSS) were compared among patients who were treated using PN, CRA, or RFA. The Cox proportional hazards model was used to determine prognostic factors for survival.

In patients with RCCs sized 2 to 4 cm, better OS and CSS were observed with PN than with CRA or RFA. On multivariable analysis, compared to PN, CRA and RFA were independently associated with poor OS and CSS in patients with RCCs sized 2 to 4 cm. In patients with RCCs sized ≤2 cm, better OS was observed with PN than with CRA or RFA; however, CSS was similar. On multivariable analysis, compared to PN, RFA was independently associated with poor OS in patients with RCCs sized ≤2 cm.

CRA or RFA should not be recommended for patients with RCCs sized 2 to 4 cm; PN is an effective treatment modality in these patients. For patients with RCCs sized ≤2 cm, CRA can be an equally effective alternative to PN.

## 1 Introduction

The incidence of small renal masses (SRMs) sized ≤4 cm has been increasing in the last 10 years; an increase in the use of imaging studies may be at least partly responsible for this increasing incidence.^[1,2]^ Partial nephrectomy (PN) has long been the gold standard for treating SRMs. Due to the increased incidence of SRMs and evolution of treatment recommendations, thermal ablative (TA) techniques, mainly cryoablation (CRA) and radiofrequency ablation (RFA), are used as alternatives to PN due to low rates of associated complications and renal function loss.^[3,4]^ Furthermore, the use of TA techniques has been confirmed as a valid approach for patients with advanced age or for those with a solitary kidney.^[5,6]^

With the increase in the use of CRA and RFA for SRMs, the debate on the superiority of CRA or RFA over PN has continued, and the best treatment option among RFA, CRA, and PN for T1aN0M0 renal cell carcinoma (RCC) is still unclear. A study reported that, in patients with tumors sized ≤2 cm, the survival rates associated with TA techniques are similar to those associated with PN.^[7]^ Another study reported that, in patients with tumors sized 2 to 3 or 3 to 4 cm, a significant difference was observed among survival rates associated with different initial local treatment modalities (observation, ablation, PN, and RFA).^[8]^ Therefore, T1a (≤4 cm) may be subclassified into T1a_1_ (≤2 cm) and T1a_2_ (2–4 cm) based on significantly different survival rates associated with different treatment modalities. To the best of our knowledge, no previous study has attempted to determine the optimal treatment procedure for patients with RCCs sized ≤2 cm or 2 to 4 cm. Herein, we used the large Surveillance, Epidemiology, and End Results (SEER) database to compare treatment modalities for T1aN0M0 RCC, with a focus on subgroups classified by tumor size (≤2 cm and 2 to 4 cm).

## 2 Materials and methods

### 2.1 Data source

The SEER database, maintained by the US National Cancer Institute, contains demographic information and data regarding cancer incidence and survival from 17 population-based cancer registries. In this study, the dataset was released in April 2016, which covered approximately 30% of the US population. The SEER database is an open access database, and data are available for research purposes. We obtained permission to access the data files with the reference number 12948-Nov2015. Since the data extracted from this database were anonymized and de-identified prior to release, our research did not require patient informed consent. The study was granted exempt status by the ethics committee of the Harbin 242 Hospital (Harbin, China).

### 2.2 Study population and variables

**Figure 1:**
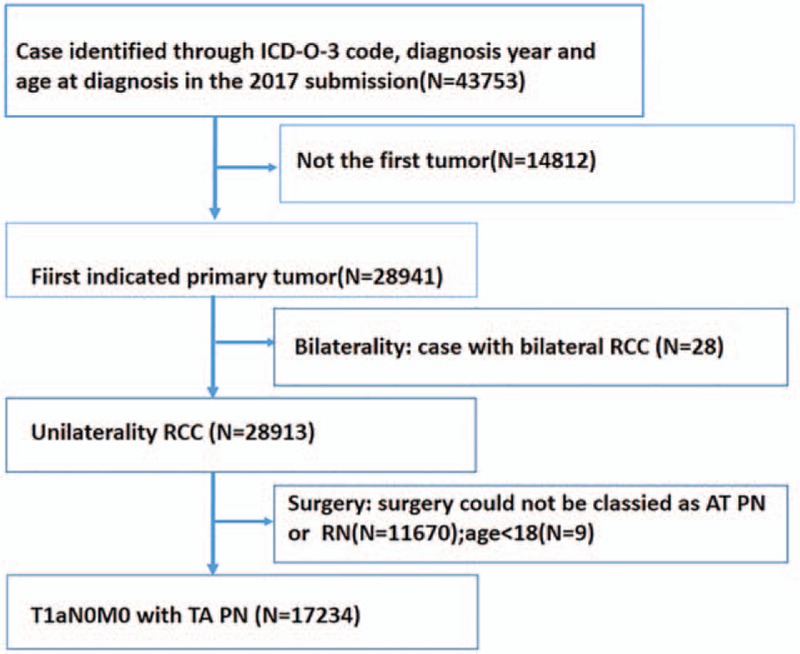
Diagram showing the process of patient selection for statistical matching.

Anonymous patients who were diagnosed with pathologically proven T1aN0M0 RCC using the latest American Joint Committee on Cancer staging system were included. Patients with renal tumors were analyzed based on the International Classification of Diseases for Oncology, Third Edition (code C64.9 and C65.9) criteria, which defines renal tumors as those with the “kidney and renal pelvis” as the primary tumor location. All patients were diagnosed between 2004 and 2015. We excluded patients aged <18 years at the time of diagnosis, those without age-related information, and cases involving autopsy or death certificates. Patient and tumor-related information included data about age, sex, race, marital status, histologic type, tumor size, tumor grade, laterality, and therapies. We limited the sample set to patients who were treated using TA techniques (RFA [SEER code 15] and CRA [SEER codes 13 and 23]) or PN (SEER code 30). Figure 1 summarizes the process of patient selection before statistical matching.

### 2.3 Statistical analysis

We used the χ^2^ test for analyzing baseline characteristics of patients with different marital status. The Kaplan–Meier method and log-rank test were used for analyzing each prognostic factor of CSS and OS, respectively. Moreover, univariate and multivariate Cox regression models were used to estimate the hazard ratio (HR) and precise 95% confidence intervals (CIs). All statistical tests were performed using SPSS 21 software (Chicago, IL). Finally, all tests were 2-sided, and the significance level was set at *P* < .05.

## 3 Results

### 3.1 Patient and tumor baseline characteristics

Overall, we identified 17,234 patients with T1aN0M0 RCCs (sized ≤4 cm); of these, 15,395 and 1839 patients were treated using PN and TA techniques, respectively. In the TA subgroup, 1381 patients underwent CRA whereas 457 underwent RFA. The median follow-up time was 30 months in both the PN and TA subgroups. The patient and tumor baseline characteristics are listed in Tables 1 and 2, respectively. Notably, surgeons were more likely to perform TA in the RCC sized 2 to 4 cm subgroup than in the RCC sized ≤2 cm subgroup.

**Table 1:**
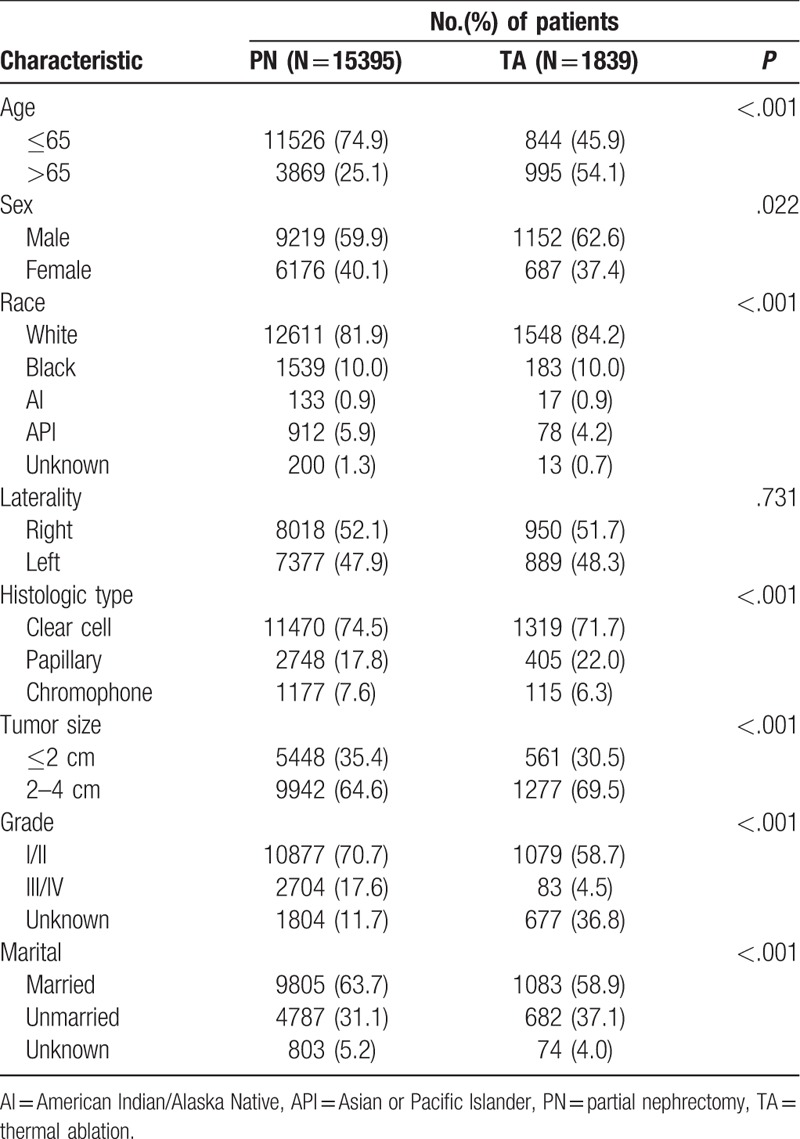
Baseline characteristics of T1aN0M0 renal cell carcinoma patients.

**Table 2:**
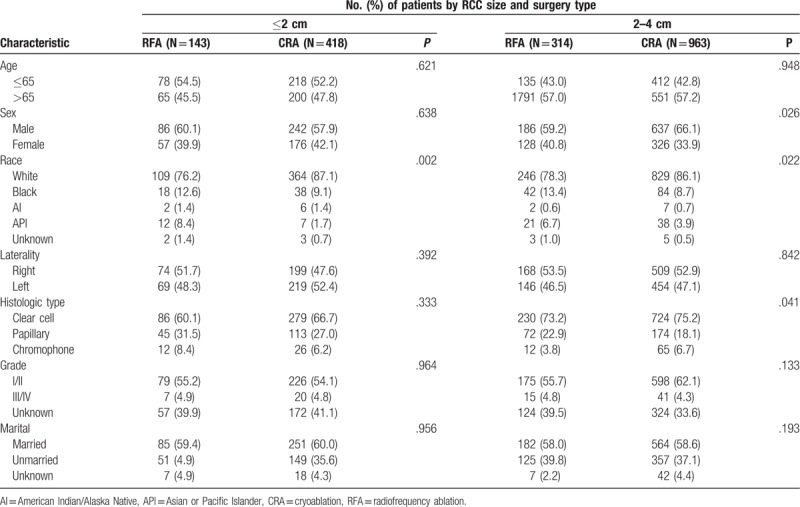
Baseline characteristics of patients with renal cell carcinoma ≤ 2 cm and 2–4 cm.

### 3.2 OS and CSS analyses in the RCC T1a sized ≤2 cm and sized 2 to 4 cm subgroups

**Figure 2:**
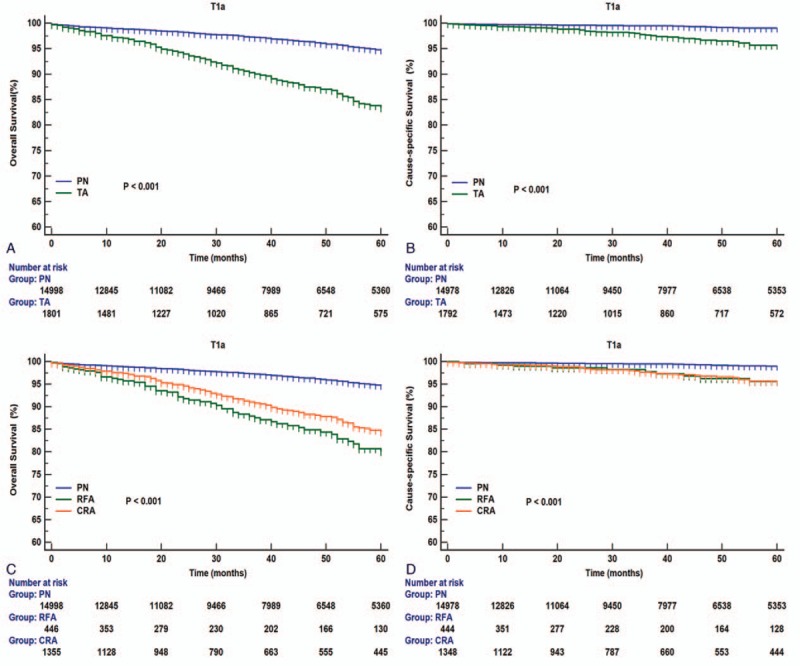
Overall and renal cancer-specific survivals in patients with T1aN0M0 renal cell carcinoma (RCC) undergoing PN or TA treatment (A and B); CRA, RFA, or PN (C and D). CRA = cryoablation, PN = partial nephrectomy, RCC = renal cell carcinoma, RFA = radiofrequency ablation, TA = thermal ablation.

**Figure 3:**
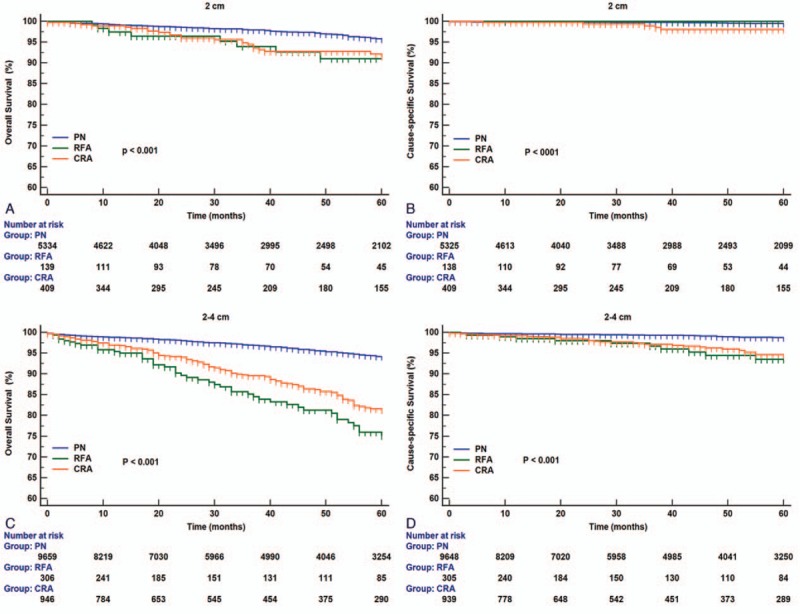
Overall and renal cancer–specific survivals in patients with renal cell carcinoma (RCC) sized ≤2 cm (A and B) or RCC sized 2–4 cm (C and D) undergoing CRA, RFA, or PN. CRA = cryoablation, PN = partial nephrectomy, RCC = renal cell carcinoma, RFA = radiofrequency ablation, TA = thermal ablation.

Results of the log-rank test showed that in T1aN0M0 RCC patients, better OS (HR, 3.247; 95% CI, 2.632–4.005; *P < *0.001) and CSS (HR, 3.481; 95% CI, 2.184–5.551; *P < *0.001) were observed with PN than with TA techniques (Fig. 2A and B). Better survival in terms of OS (PN vs. CRA: HR 2.995, 95% CI 2.363–3.794, *P < *0.001; PN vs. RFA: HR 4.085, 95% CI 2.683–6.220, *P < *0.001) and CSS (PN vs. CRA: HR 3.562, 95% CI 1.399–6.220, *P < *0.001; PN vs. RFA: HR 3.457, 95% CI 2.043–5.850, *P < *0.001) was observed in the PN subgroup than in the CRA or RFA subgroups (Fig. 2C and D). For RCCs sized ≤2 cm, a difference was observed in OS (PN vs. CRA: HR 1.958, 95% CI 1.204–3.184, *P < *0.001; PN vs. RFA: HR 2.841, 95% CI 1.211–6.662, *P < *0.001) but not in CSS (Fig. 3A and B). According to the RCC subclassification, in patients with RCCs sized 2–4 cm, survival outcomes differed significantly according to treatment modality with respect to OS (PN vs. CRA: HR 3.284, 95% CI 2.513–4.292, *P < *0.001; PN vs. RFA: HR 4.497, 95% CI 2.782–7.269, *P < *0.001) and CSS (PN vs. CRA: HR 3.536, 95% CI 2.006–6.234, *P < *0.001; PN vs. RFA: HR 4.339, 95% CI 1.573–11.971, *P < *0.001) (Fig. 3C and D).

The Cox proportional hazards regression model was used to control for potential confounding factors. Compared to TA techniques, PN was significantly associated with better OS (HR, 2.327; 95% CI, 1.953–2.772; *P < *0.001) and CSS (HR, 2.174; 95% CI, 1.502–3.147; *P < *0.001) for RCCs sized 2–4 cm but not for RCCs sized ≤2 cm (OS: HR 1.489, 95% CI 1.064–2.084, *P = *0.020; CSS: HR 1.248, 95% CI 0.504–3.092, *P = *0.632; Table 3).

**Table 3:**
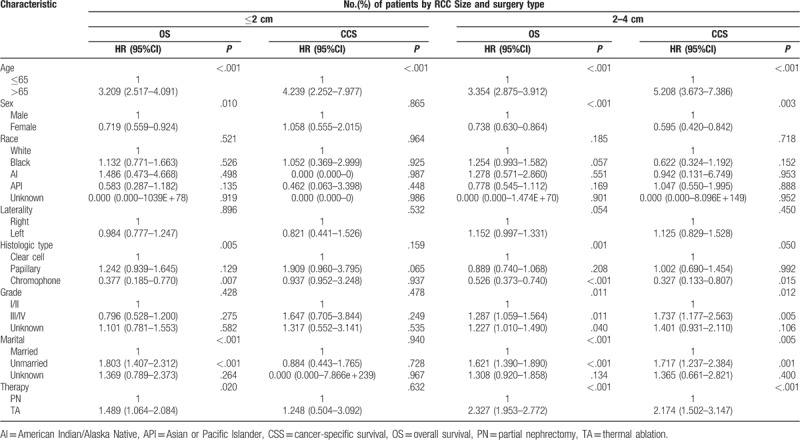
Cox proportional hazards regression model for overall survival and renal cancer-specific survival in patients with renal cell carcinoma ≤ 2 cm and 2–4 cm who underwent thermal ablation and partial nephrectomy.

Furthermore, our results showed the superiority of PN over CRA and RFA for RCCs sized ≤2 cm and RCCs sized 2–4 cm. In patients with RCCs sized 2–4 cm, worse OS (PN vs. RFA: HR 2.988, 95% CI 2.263–3.946, *P < *0.001; PN vs. CRA: HR 2.144, 95% CI 1.766–2.603, *P < *0.001) and CSS (PN vs. RFA: HR 2.626, 95% CI 1.441–4.786, *P = *0.002; PN vs. CRA: HR 2.051, 95% CI 1.368–3.075, *P = *0.001) were observed with RFA and CRA than with PN (Table 4). In patients with RCCs sized ≤2 cm, worse OS was observed with RFA than with PN (HR, 2.034; 95% CI, 1.172–3.531; *P = *0.012); however, this difference was not observed between PN and CRA (HR, 1.326; 95% CI, 0.897–1.960; *P = *0.157). In patients with RCCs sized ≤2 cm, no significant differences were observed in CSS between RFA or CRA and PN (PN vs. RFA: HR 0.000, 95% CI 0.000–5.163, *P = *0.986; PN vs. CRA: HR 1.699, 95% CI 0.682–4.234, *P = *0.255; Table 4).

**Table 4:**
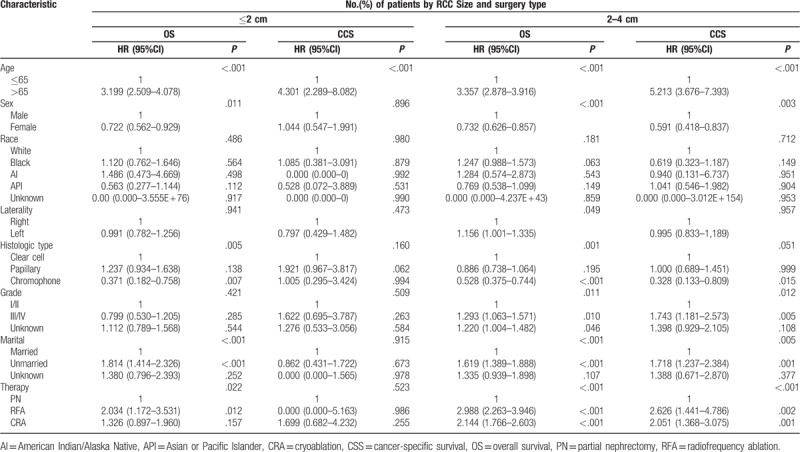
Cox proportional hazards regression model for overall survival and renal cancer-specific survival in patients with RCC ≤ 2 cm and 2–4 cm who underwent partial nephrectomy, radiofrequency ablation, and cryoablation.

## 4 Discussion

Several novel approaches have been applied in the treatment of SRMs sized ≤4 cm; however, the optimal treatment of SRMs remains controversial. Recently, several reports have suggested comparable oncologic outcomes between TA techniques and PN in RCC patients.^[9–11]^ However, these studies have not presented substantial or compelling evidence in favor of TA techniques; therefore, PN is still the standard treatment for SRMs and is recommended by the European Association of Urology as well as the American Urological Association.^[12,13]^ Although these recent technologies present obvious advantages for patients, the debate still continues about whether CRA or RFA could be an oncologic treatment equivalent to PN for SRMs. For this study, we classified T1aN0M0 RCC into subgroups based on tumor size: ≤2 cm and 2 to 4 cm. We aimed to study whether any difference exists among RFA, CRA, and PN as treatment modalities for both subgroups. In our study, we demonstrated that PN was superior to CRA or RFA in patients with RCCs sized 2 to 4 cm. However, in the RCC size ≤2 cm subgroup, no obvious superiority was found between PN and CRA. Using this subclassification, we aimed to provide a new basis for stratifying patients and for targeting SRMs that are most likely to be cured in an optimal and durable manner.

Two SEER-based studies and one meta-analysis evaluated the difference in survival rates between PN and TA techniques for SRMs.^[9,14,15]^ All studies showed similar results: the PN group showed significantly better OS, but no significant difference in CSS was observed between the treatment groups. In the light of these findings, we aimed to provide more comprehensive information for identification of the optimal treatment strategy. Therefore, we performed this population study to assess how treatment with TA techniques and PN correlated with the reported findings. Initially, our study showed that, compared to TA techniques, PN had a significant survival (OS and CSS) benefit in patients with T1aN0M0 RCCs. Subsequently, TA was divided into CRA and RFA, and patients were further divided into subgroups according to tumor size, ≤2 cm and 2 to 4 cm. We found that significantly worse OS and CSS was observed with CRA or RFA compared with PN in patients with RCCs sized 2–4 cm. This relationship was not observed in those with RCCs sized ≤2 cm. Therefore, we hypothesized that variables such as tumor size and treatment modality significantly contribute to survival. Based on this difference in survival outcomes, T1a (≤4 cm) tumors should be classified into 2 subgroups according to a 2-cm cutoff. Moreover, a study by Abdel-Rahman et al^[8]^ supported the idea that T1a lesions should be divided into T1a_1_ and T1a_2_ according to tumor size (using 2 cm as a cutoff value). This study demonstrated that PN, instead of CRA or RFA, is the preferred choice in patients with RCCs sized 2 to 4 cm for providing optimal long-term survival. This result is consistent with National Comprehensive Cancer Network treatment guidelines, which recommend PN for lesions sized > 3 cm; these guidelines recommend TA techniques for select patients with small lesions, for older patients, and for patients with competing health risks.^[16]^

In clinical practice, some patients are not eligible for PN owing to co-morbidities or older age.^[3]^ In such cases, TA is a feasible alternative as it has the advantages of a short hospitalization period, reduced morbidity, and tolerability in patients with significant co-morbidities.^[17]^ CRA and RFA are both standard TA techniques; however, it is still unclear which technique has the maximum oncologic efficiency. In 2 retrospective studies, a comparison between CRA and PN revealed similar OS, CSS, recurrence-free survival (RFS), and metastases-free survival (MFS).^[18,19]^ Conversely, 2 other retrospective studies reported better oncologic outcomes with PN than with CRA.^[20,21]^ A recent study at the Mayo Clinic revealed similar RFS rates between PN and CRA but reported better MFS with PN and CRA than with RFA.^[6]^ More recently, a retrospective matching study revealed that TA techniques and PN resulted in similar OS in patients with T1a tumors sized ≤2 cm.^[7]^ In our study, which is based on a large cohort, we could obtain a more refined result. In patients with RCCs sized ≤2 cm, multivariable analyses showed that, except for a difference in OS between the PN and RFA groups, no difference was observed in OS and CSS between the PN and TA groups. Despite this disparity between earlier and more recent studies, our results together with existing data suggest that TA techniques, specifically CRA, could be a feasible alternative to PN, especially for SRMs ≤2 cm.

However, our study has certain limitations. First, as is common in retrospective studies, many inherent biases could not be avoided. Selection bias due to the absence of some important patient characteristics, such as functional status, could not be disregarded. Second, we could only obtain information regarding initial treatment from the SEER database; no information was available regarding subsequent treatment after relapse. This may have affected our findings. Third, the SEER database also lacks other information such as comorbidities, number of tumors, proximity of the tumor to other structures, and type of surgery (open, laparoscopic, or robotic-assisted). These factors are associated with varying degrees of morbidity and therefore, affect patient survival. Despite these limitations, our results have provided some indications for elucidating the optimal management strategy in patients with SRMs. Further investigation, ideally in the form of a randomized controlled trial, is needed.

In conclusion, significantly better survival was observed with PN compared with TA (CRA or RFA) techniques in patients with RCCs sized 2 to 4 cm. CRA or RFA should not be recommended for patients with RCCs sized 2 to 4 cm; PN is a better alternative in such patients. However, for patients with RCCs sized ≤2 cm, CRA can be an equally effective alternative to PN. Further prospective randomized studies are needed to evaluate this treatment paradigm for T1aN0M0 RCC.

## Author contributions

Shuai Yan: Project development, data collection and management, and data analysis; Wei Yang: Manuscript writing and editing; Cheng-mei Zhu and Pei-meng Yan: Project development and manuscript writing and editing; and Zhi-chao Wang: Data analysis and manuscript writing.

**Formal analysis:** Wei Yang.

**Methodology:** Cheng-mei Zhu.

**Project administration:** Zhichao Wang.

**Software:** Cheng-mei Zhu.

**Supervision:** Zhichao Wang.

**Writing – original draft:** Shuai Yan, Wei Yang.

**Writing – review & editing:** Pei-meng Yan.
